# Normality in medicine: an empirical elucidation

**DOI:** 10.1186/s13010-022-00127-z

**Published:** 2022-12-21

**Authors:** Michael Rost, Maddalena Favaretto, Eva De Clercq

**Affiliations:** Institute for Biomedical Ethics, University of Basel, Bernoullistr. 28, 4056 Basel, Switzerland

**Keywords:** Normality, Normativity, Mental health, Physical health, Authority, Injustice, Discrimination, Medicine

## Abstract

**Background:**

Normality is both a descriptive and a normative concept. Undoubtedly, the normal often operates normatively as an exclusionary tool of cultural authority. While it has prominently found its way into the field of medicine, it remains rather unclear in what sense it is used. Thus, our study sought to elucidate people’s understanding of normality in medicine and to identify concepts that are linked to it.

**Methods:**

Using convenient sampling, we carried out a cross-sectional survey. Since the survey was advertised through social media, we employed an online survey. We performed descriptive and inferential analyses. Predictors were chosen in a theory-driven manner.

**Results:**

In total, 323 persons from 21 countries completed the survey. Analysis revealed that the overall acceptance of normality in medicine was associated with notions of injustice, authority, discrimination, and with having a medical profession. More precisely, for the field of mental health, injustice insensitivity, genderism and transphobia, and authority were positively associated with a person’s acceptance of normality; and, for the field of physical health, injustice insensitivity and having a medical profession were positively associated with a person’s acceptance of normality. Finally, participants’ acceptance of the use of normality in the area of mental health was lower than in the area of physical health.

**Conclusions:**

What is considered normal has implications for clinical practice, both at an individual and at a policy-level. Acknowledging its normalistic condition, the discipline of medicine has to confront itself with its own contribution to the augmentation of social inequalities through the excessive reliance on the concept of normality. Research that centers the lived experiences of those who are being systematically marginalized because they are deemed abnormal is needed. By empirically elucidating the conceptual relationships between normality in medicine and other variables, we provide points of leverage to deprive normality of its normative power. For medicine, this is needed to first do no harm.

**Supplementary Information:**

The online version contains supplementary material available at 10.1186/s13010-022-00127-z.

## Introduction

The normal is nowadays taken for granted, but it has only become culturally ubiquitous since the middle of the twentieth century [1, 2]. Before, the term was primarily used in professional settings (e.g. statistics) where it referred to a range of variations, in particular to a symmetric, bell-shaped distribution of data in which most data fall near the center. Consequently, “normal” was often used as a synonym for the “probable” and did not yet describe “one part of a binary condition in which the other term is the deviant, the pathological, the abnormal” ([2],p15). This has changed completely and today, people adhere to the normal both as a normative (i.e. prescribing how the world or a person ought to be) and a descriptive concept (i.e. indicating how the world or a person is) in societal as well as in professional contexts. Recent psychological research, in fact, has evidenced that people’s notion of normality generally incorporates both statistical (e.g. average) and prescriptive (e.g. the ideal) norms, that is both an object’s occurrence probability and its goodness influence a person’s normality evaluation [3, 4].

The normal has prominently found its way into the field of medicine. Already in 1953, the British psychologist Eysenck noted that normality “recurs with disturbing frequency in the writings of psychologists, psychiatrists, psychoanalysts, and sociologists” ([5],p177). A few years later, the French philosopher and physician Canguilhem expressed concerns about the medical trend of defining the normal in mere statistical terms. He argued for “the logical independence of the concepts of norm and average” and against “producing the full equivalent of the anatomical or physiological normal in the form of an objectively calculated average” ([6],p155/156). Despite Canguilhem’s unease about the term and its statistical foundations, “normal” has increasingly been used in the medical setting. This becomes apparent if we look at the two internationally authoritative guides for mental and physical disorders, respectively, the Diagnostic and Statistical Manual of Mental Disorders (DSM) and the International Statistical Classification of Diseases and Related Health Problems (ICD) [7].

While the term “normal” appeared only 19 times in the DSM 1 (1952), in the most recent edition (at the time of data collection), DSM-5 (2013) it is present 366 times (e.g. normal level of intellectual functioning, abnormality of emotional processing, normal sexual desire, normal pattern of learning academic skills). Correspondingly, in the ICD-10 Classification of Mental and Behavioural Disorders (1992) it appeared 259 times (e.g. normal mood, normal family, normal sense of (fe)maleness, normal children). Finally, in the ICD-11 (2020) it is used 1445 times (e.g. normal delivery, normal range of life experiences, normal skin, normal grief, abnormal social behaviour, normal weight, normal personality characteristics). Hence, for both mental and physical health, the normal is an important diagnostic category for practitioners worldwide. Still, until today, it remains rather unclear in what sense “normal” is used in medicine [8]: in disease classification systems, for example, does it refer to a value-free biostatistical model of normality, to a value-laden normative model of normality, or maybe simply to health? And if its meaning remains unclear, then how can it provide any concrete guidance in medical practice?

Several attempts have been made to identify and classify the various uses of the concept in medicine. In 1958, surgeon Marvin Wellman identified two opposite meanings, namely the normal as what is most usual and as the perfect and ideal. Between these two extremes numerous different significations of the normal exist (e.g. health, value to the individual, functioning in accordance with structure, all data points fall under the bell curve and thus are normal) [9]. A recent review identified five meanings of normality in medicine: a biostatistical theory, health, an ideal, a process, and a biological advantage [8]. Furthermore, it stressed the importance of redesigning the concept of normality in medicine according to current times, for example it has to address diversity [8]. Both articles thus illuminate that normality in medicine is a hybrid composed of a descriptive (e.g. statistics, averages) and a normative (e.g. value judgement, the ideal) part.

The centrality and normativity of normality have been the subject of critical research, amongst others, in the fields of disability studies, gender studies, anthropology, psychiatry, medical humanities, history and philosophy of medicine, cultural studies, sociology, and critical race studies [2]. Applying a critical lens to the concept of the normal, much of these scholarly projects have focused on the cultural authority and the potential dangers of the concept of and an (uncritical) acceptance of normality. Broadly speaking, they found that, at a societal level, “the normal functions as a hidden system of compulsory conformity” and, at the individual level, “this unassuming word [normal] can have a significant effect on the lives of those defined in contrast to it as abnormal, pathological, or deviant.” (2,p2,6) The seemingly neutral label “normal” reinforces a certain worldview, a set of human behaviors and qualities, and risks to perpetuate systems of privilege and power [2]. As such the use of “normal” tends to marginalize, stigmatize, pathologize, or discriminate against those deemed different and amplifies existing inequalities because it suggests that there is something as an objective normality. By doing so, it often operates normatively as an exclusionary tool of cultural authority, as illustrated by the following findings from medicine and other disciplines. It has been demonstrated (and criticized) that the idea of.

- a *normal body* leads to oppressive narratives about persons with physical disabilities [10], results in a desire for bodily normalcy [11, 12], and is used to assimilate indigenous people into the nation-state [13];
- *normal hearing abilities* imposes normative assumptions upon Deaf people [14];
- the *normal brain* risks to pathologize and (dis-)qualify individual human brains as abnormal [15], to legitimize status quo educational practices[16], and to augment social inequalities [17];
- *normal sexuality* (i.e. heterosexuality) is instrumentalized as a political norm crucial for being a full member of society [18];
- *normal sexes* (i.e. binary) threatens the bodily integrity of intersex people [19];
- *normal characteristics of children* (e.g. ethnic, religious, racial, bodily, social-economic) tends to marginalize children defined as “different” [20];
- a *normal social identity* forces religious minorities to perform acts of ideal adjustment to the majority [21];
- a *normal family* renders “less traditional” ones invisible [22];
- *normal skin color* stigmatizes any person with a different skin color as an outsider [23, 24];
- *the normal in medicine* mistakenly suggests that normality does have an objective existence [17, 25];
- the *idea of biological normality* leads to implicit social judgements about the acceptability of certain kinds of biological variation [26]
- a *statistical concept of normal* is a mechanism of power and control [27].

With respect to these examples, social psychological research suggests that being considered less normal is indeed associated with lower collective self-esteem, and experiences of stigmatization and disempowerment [28, 29]. To conclude with the words of the Canadian philosopher Ian Hacking, “the benign and sterile-sounding word ‘normal’ has become one of the most powerful ideological tools of the twentieth [and twenty-first] century” ([30],p169).

To our knowledge, however, this is the first study to quantitatively examine the conceptual relationships between normality and other possibly related variables as viewed by either lay people or health professionals. Little is known about which dispositional variables are associated with a person’s acceptance of normality or which particular normative concepts – in people’s representations – appear to be related to normality after all. We know that there is a moral dimension to the concept of normal, but what features of normality, both in theory and in (medical) practice, render it a normative category? As such, the purpose of this study was to connect some conceptual dots surrounding the use of normality by regressing a person’s acceptance of normality as being applied in medicine on theoretically derived predictor variables. Our analysis did not focus on how people determine normality, what type of norms they incorporate, or how normality has developed over the centuries. It tackles the question of which socio-demographic, personality-related, or morality-related variables go hand-in-hand with normality in people’s minds in real-world-settings. Thus, this study sought to (a) elucidate people’s (lay persons’ and medical professionals’) understanding of normality in medicine and, based on this, to (b) identify concepts that are, at least in people’s implicit conceptualizations, linked to the concept of normality. Filling the conceptual vacuum around normality is central to dismantling its normative power and to, ultimately, working towards a world free of discrimination.

## Methods

### Study design and ethical approval

This quantitative study is part of a larger social media project on the use of normality in medicine and presents the analysis of an English cross-sectional online survey. The survey was hosted on the free German online survey site soscisurvey.de. Since data was collected anonymously, the study did not fall under the remit of the Swiss Human Research Act (Art. 2). Hence, no ethical approval was needed. Nevertheless, the responsible ethics committee issued a declaration of no objection. It stated (EKNZ; Req-2020–00,292) that the project fulfills the general ethical and scientific standards for the research with humans (see Art. 51, Swiss Human Research Act). Furthermore, the University’s data protection office approved the online survey tool used and anonymity of data collection.

### Survey construction

Survey construction involved several steps. First, the research team discussed potential dependent and independent variables, that is the operationalization of normality in medicine and possible predictors of the latter. Related to this, it has to be noted that there is a paucity of empirical studies systematically evaluating the conceptual relationships between normality in medicine and other variables, such as psychological or morality-related constructs. Still, intra-team discussions on variables possibly associated with normality were based on (a) the research team’s knowledge in the field of bioethics, (b) existing theoretical work from other disciplines (e.g. sociology, philosophy of medicine, linguistics, queer theory, disability studies) which has focused on the relationships between norms, normality, and normativity, and (c) a systematic literature review on normality in connection with intersex [31]. Second, existing validated questionnaires were identified that captured variables which were agreed upon in the previous step. Third, identified questionnaires were evaluated based on their psychometric quality criteria such as reliability (i.e. precision of measurement) and content validity (i.e. the extent to which their constructs capture the theoretically derived concepts, possibly related to normality in medicine). Questionnaires with low reliability or content validity were excluded. Fourth, questionnaires that were deemed most suitable were compiled into the study survey. Accordingly, team members’ proposals on the selection of variables were converged and reconciled by achieving consensus through discussions (step one), limited by the availability of validated and suitable tools (step two and three) and statistical considerations surrounding sample-size/predictor ratio for regression analyses. Lastly, the survey was pilot tested by 10 persons. This led to minor changes of the survey structure. The included question(naire)s can be grouped into five categories: (a) demographics (e.g. age, profession), (b) personality-psychological constructs (e.g. self-esteem, Big Five), (c) morality-related psychological constructs (e.g. injustice sensitivity, authority), (d) quality of life (e.g. critical life events, general health), and (e) the dependent variable, namely normality in medicine (Table 1).

**Table 1:** Possible predictors

	**Predictors**	**Content**	**Rationale/Hypotheses**
Socio-demographics and religious/political attitudes	Age, gender, country, education, profession	- Person’s background - Human Development Index [32] used for country	- Sample description - Possibly associated with normality, especially a medical profession [8, 15–17, 27, 33]
	Religiosity [34]	- Tendency of how religiously a person assesses him/herself	- Religious persons might be more receptive to normative proto-normalistic positions [35, 36]
	Left–right self-placement [37]	- Political attitudes on a left–right dimension	- Normality in medicine and normal sexuality are highly political and the normal has political implications [13, 18]
Personality-psychological constructs	Big Five Inventory [38]	- Dimensions of personality: Openness, Conscientiousness, Agreeableness, Extraversion, Neuroticism	- Personality dimensions might affect a person’s acceptance of normality (e.g. as some sort of status quo) [39, 40] - Relationships between personality, prejudice, and ideological attitudes [41]
	Self-esteem [42]	- Self-esteem of participants	- “Less normal” minority groups might have lower self-esteem [28, 40]
	Tolerance for Ambiguity [43]	- Dimensions of tolerance for ambiguity: valuing diverse others, change, challenging perspectives, unfamiliarity	- Persons in situations of ambiguity and under uncertainty or who are less tolerant for ambiguity might be more likely to subscribe to the normal [44, 45]
Morality-related constructs	Moral Foundations [46]	- Individual priorities in moral reasoning; dimensions: care and harm, fairness and cheating, loyalty and betrayal, authority and subversion, sanctity and degradation	- Persons’ accounts of normality are part prescriptive and part descriptive, they combine information about an ideal and an average – that means normality is a normative concept [3, 4]
	Injustice Sensitivity [47]	- Psychological characteristic of injustice sensitivity; subscales: victim’s, observer’s, perpetrator’s, and beneficiary’s perspective	- It has been argued that the normal stigmatizes, marginalizes, pathologizes, and discriminates against individuals who are defined in contrast to it [2, 11, 14, 17, 22]
	Genderism and transphobia [48]	- Negative attitudes and propensity for violence toward trans* people	- Sexuality and gender identity might be associated with an idea of normality [11, 18, 49] - Discrimination against and negative attitudes towards non-binary people [50, 51]
Qualityof life	Subscale of SF-36: General health [52]	- Overall self-rated health	- It has been argued that normality becomes more important in times of crisis (e.g. disease) [35, 36]
	Critical life events [53]	- Critical life events in the past year	- It has been argued that normality becomes more important in times of crisis (e.g. critical life events) [35, 36]

Survey construction requires researchers to critically reflect on underlying assumptions and researcher positionality. The research team’s backgrounds and expertise span Psychology, Bioethics, Philosophy, Anthropology, Cultural Communication, and Feminist Theory. We are committed to using research to uphold human rights (e.g. right not be discriminated against) and to contribute to change. In particular, we aim to better understand the underlying drivers of social inequality. We acknowledge that our analysis is situated within a Euro-Western context. Researchers’ formative life experiences include: personal loss; being white and cisgender; privilege in education and personal safety; and severe health conditions.

### Sampling and data collection

The online survey was advertised through social media (Twitter, Facebook) and through the local university’s online flea market as part of the larger social media project between May and November 2020. Project funding did not include financial resources for probabilistic sampling methods. Hence, we employed an online convenient sampling method. Furthermore, to purposely oversample persons with a medical background, we sent the survey to professional contacts of the research team. The name for the respective social media channels was “BanAnyNormality?”. The purpose of the larger social media project was to make the normal an object of personal reflection and to stimulate a critical discussion by posting diverse content (e.g. research and newspaper articles, videos) on the subject of normality in various fields (e.g. arts, science, politics).

### Study population and study sample

All persons aged 18 or older could participate in the survey. However, access to internet and proficient English language skills were required. We cannot estimate the response rate. Completion rate was 88.7% (323/364).

### Dependent variable(s)

At the individual level, the conceptual use of normality (in medicine) has not been studied empirically. Our study’s objective was to address this research gap. Thus, we had to first operationalize normality in medicine. For this purpose, we created a 12-item scale that captured a person’s acceptance of normality in medicine. For each item, we used an example of the term “normal” from ICD-11 or from DSM-5. We used six examples from ICD-11 (normal pregnancy; abnormality of gait and mobility; normal physiological development; abnormal bowel sounds; normal head movements; abnormality of heartbeats) and six examples from DSM-5 (normal fluency and time-patterning of speech; abnormality of beliefs, thinking, and perception; normal pattern of learning academic skills; abnormal social approach; normal level of intellectual functioning; abnormality of emotional or cognitive processing); thereby creating two subscales (r = 0.579, p = 0.000), namely a person’s acceptance of normality in the areas of physical (ICD-11) and mental health (DSM-5). For each subscale, we used three examples of “normal” and three examples of “abnormal”.

For all 12 items, we asked the participants to “indicate [their] opinion about the following statements. Do you think there is such thing as: [followed by the 12 examples]?”. We used a 5-point Likert scale (1 = strongly disagree; 2 = disagree; 3 = neither disagree, nor agree; 4 = agree; 5 = strongly agree). The three scales (overall, mental, physical) were calculated by summing up the item scores. Thus, the overall scale ranged from 12 to 60 and the two subscales ranged from 6 to 30. The scales’ reliability as indicated by internal consistency was high (αoverall = 0.854; αmental = 0.812; αphysical = 0.751; item-total-correlations in appendix). By reliably measuring a person’s acceptance of normality in medicine, these scales can be interpreted as a person’s (dis)approval of (the ontological existence of) normality in medicine. They ascertain the extent to which a person thinks normality in medicine exists. Therefore, they lend themselves to the elucidation of the relationships between normality in medicine and other variables without necessitating a positive definition of normality in medicine. Possible associations with other variables could thus be interpreted as implicit conceptualizations surrounding normality in medicine on the part of the person.

Determining conceptually related variables (through regression analyses) was meant to enable us to shed light on the conceptual vacuum around normality in medicine, that is by identifying convergent and discriminant (related to each other to a high or small degree, or not at all) concepts. It is particularly this conceptual use of normality in medicine which our study addressed empirically for the first time.

### Independent variables

As described earlier, possible predictors were chosen based on the question of which other variables explain a person’s (dis)approval of normality in medicine? Since, to our knowledge, there are no empirical studies on the conceptual relationships between normality in medicine and other variables, we selected possible predictors based on existing theoretical work on normality and the research team’s knowledge. Apart from sociodemographic variables, we exclusively used validated measurement scales. Our hypotheses- and theory-driven rationale for selecting independent variables is summarized in Table 1.

### Statistical analyses and predictor selection

We used SPSS 26.0 to quantitatively analyze the data. First, we performed descriptive analyses. Second, independent factors associated with people’s acceptance of normality in medicine were determined using multiple linear regression analysis. In addition, moderator analyses were conducted. Statistical significance level was set at *p* < 0.05. For multiple linear regression analysis, assumption checks were performed before interpretation of the model (see appendix). Besides theoretical considerations, sample-size/predictor ratio a priori determines variable selection for regression modeling. According to Harrell, a fitted regression model is likely to be reliable when *p* < *m*/15, where *p* is the number of predictors and *m* is the sample size [54]. Applying this requirement to our sample size (*N* = 323) and anticipating missing data, we limited the number of included predictors to 20.

In total, we selected 23 predictors: five socio-demographic variables, two variables capturing political and religious attitudes, seven personality-psychological variables, seven morality-related variables, and two variables capturing quality of life as possible predictors (Table 1). Gender (possible responses: woman, man, non-binary/third gender, prefer to self-describe, prefer not to say) could not be meaningfully recategorized without losing too much data and, thus, was not included in the regression models. To further limit the number of predictors we excluded a person’s left–right self-placement, because this continuum can be captured by the included moral foundations scales [55]. We also excluded the education variable (possible responses: university degree – yes/no), because this variable was partially captured by the question on a person’s profession.

The following 20 predictors were included in the regression models: age, the Human Development Index (HDI) of a person’s country, profession, religiosity, the Big Five subscales (Openness, Conscientiousness, Agreeableness, Extraversion, Neuroticism), self-esteem, tolerance for ambiguity, the five Moral Foundations subscales (care/harm, fairness/cheating, loyalty/betrayal, authority/subversion, sanctity/degradation), injustice sensitivity, genderism and transphobia, general health, and critical life events.

## Results

### Socio-demographic characteristics of the sample and acceptance of normality

In total, 323 persons from 21 countries and five continents (Europe, Africa, North-America, Asia, Oceania) completed the survey. The mean age of respondents was 35 years. Almost six out of ten participants were women, more than two third were located in European countries, and almost four out of five held a university degree. The professional background of one out of five participants was closely related to the field of medicine and one out of six participants self-identified as strongly religious. More than nine out of ten participants assessed their political attitudes as left or centrist. Participants’ acceptance of the use of normality in the area of mental health was lower than in the area of physical health. Paired samples t-test revealed that the difference was statistically significant t(314) = -7.398, *p* = 0.000. Further socio-demographic information and descriptives for participants’ acceptance of normality in medicine are presented in Table 2.

**Table 2:** Socio-Demographics and acceptance of normality

Socio-demographics	
Age (N = 321)	M = 34.85 (SD = 10.4), Mdn = 34, Mo = 37, Min = 18, Max = 76
Gender (N = 323)	59.1% women, 35.6% men, 5.3% diverse^1^
Country (N = 321)^2^	Switzerland 29.6%, Germany 28.7%, Nigeria 16.8%, UK 4.4%, USA 3.4%, Canada 3.1%, Belgium 1.6%, Iran 1.6%, Australia 1.2%; other 9.6%
Medical background (N = 322)^3^	Not/little related [1–3]: 48.1%, moderately related [4–7]: 31.1%, closely related [8–10]: 20.8%
University degree (N = 323)	Yes: 79.9%
Religiosity (N = 323)^4^	Not/little religious [1–3]: 59.1%, moderately religious [4–7]: 24.2%, strongly religious [8–10]: 16.7%
Left–right self-placement (N = 318)^5^	Left/rather left [1–3]: 50.0%, centrist [4–7]: 41.2%, right/rather right [8–10]: 8.8%
**Acceptance of normality**	
Overall scale (N = 315)^6^	M = 41.23 (SD = 7.8), Mdn = 42, Mo = 42, Min = 12, Max = 60
Mental health (N = 319)^7^	M = 19.77 (SD = 4.7), Mdn = 20, Mo = 20, Min = 6, Max = 30
Physical health (N = 315)^7^	M = 21.46 (SD = 4.1), Mdn = 22, Mo = 23, Min = 6, Max = 30
Note*.*^1^ = non-binary, prefer to self-describe, prefer not to say, ^2^more than 1.0% of sample; ^3^10-point Likert item ranging from 1 (not at all) to 10 (entirely): “How closely is your professional background related to the field of medicine?”; ^4^10-point Likert item ranging from 1 (not religious) to 10 (religious); ^5^10-point Likert item ranging from 1 (left) to 10 (right); ^6^Possible range: 12–60; ^7^Possible range: 6–30	

### Regression analyses

Using multiple linear regression analyses, we evaluated predictors’ associations with participants’ acceptance of normality in medicine for the overall scale (regression 1) as well as for the mental and physical health subscales (regression 2, 3). Before interpreting the multiple linear regression models, assumptions checks were performed, revealing that all assumptions were met. F-test was conducted to test for the statistical significance of the overall model fits, indicating that the predictors included in the three models significantly contributed to the explanation of the acceptance of normality in medicine (Table 3).

**Table 3:** Multiple linear regression analyses

		**Regression 1** – **Overall scale** **(N = 298)**							**Regression 2** – **Subscale** **mental (N = 301)**							**Regression 3** – **Subscale** **physical (N = 298)**						
		(Un)standardized coefficients					Collinearity statistics		(Un)standardized coefficients					Collinearity statistics		(Un)standardized coefficients					Collinearity statistics	
		B	S.E	Beta	t	Sig	Tol	VIF	B	S.E	Beta	t	Sig	Tol	VIF	B	S.E	Beta	t	Sig	Tol	VIF
	Constant	30.932	9.193		3.365	.001			12.397	5.498		2.255	.025			18.761	5.102		3.677	.000		
Socio-Demographics	Age	-.058	.047	-.076	-1.230	.220	.694	1.441	-.028	.028	-.061	-.983	.327	.698	1.433	-.031	.026	-.077	-1.177	.240	.694	1.441
	Country	5.121	4.503	.097	1.137	.256	.370	2.701	4.302	2.691	.138	1.599	.111	.360	2.775	.649	2.500	.023	.260	.795	.370	2.701
	Profession	.291	.124	.128	2.353	**.019**	.902	1.109	.124	.074	.092	1.674	.095	.897	1.115	.165	.069	.139	2.408	**.017**	.902	1.109
	Religiosity	-.190	.178	-.079	-1.067	.287	.488	2.049	-.074	.107	-.052	-.690	.491	.478	2.092	-.111	.099	-.088	-1.121	.263	.488	2.049
Personality-psychologicalconstructs	Big-5: O	-.162	.262	-.034	-.620	.536	.881	1.135	-.205	.157	-.072	-1.308	.192	.881	1.135	.045	.145	.018	.310	.757	.881	1.135
	Big-5: C	.170	.278	.039	.611	.542	.675	1.480	.108	.166	.041	.652	.515	.676	1.479	.070	.154	.030	.457	.648	.675	1.480
	Big-5: E	-.335	.215	-.087	-1.560	.120	.864	1.157	-.193	.129	-.084	-1.499	.135	.862	1.161	-.147	.119	-.072	-1.233	.219	.864	1.157
	Big-5: A	-.352	.272	-.074	-1.294	.197	.814	1.229	-.131	.163	-.046	-.805	.422	.810	1.234	-.215	.151	-.086	-1.421	.157	.814	1.229
	Big-5: N	.179	.224	.049	.798	.426	.704	1.420	.095	.134	.044	.711	.477	.704	1.421	.093	.124	.049	.746	.456	.704	1.420
	Self-Esteem	.112	.109	.073	1.033	.303	.535	1.868	.048	.065	.052	.738	.461	.536	1.865	.061	.060	.076	1.013	.312	.535	1.868
	Tol. ambiguity	-.001	1.115	.000	-.001	1.00	.757	1.321	.129	.668	.011	.193	.947	.757	1.320	-.116	.619	-.012	-.187	.852	.757	1.321
Morality-related constructs	Injustice sens	-1.528	.564	-.160	-2.709	**.007**	.765	1.307	-.880	.333	-.156	-2.640	**.009**	.772	1.295	-.666	.313	-.133	-2.128	**.034**	.765	1.307
	MF: harm	.613	.781	.053	.785	.433	.595	1.681	.584	.465	.079	1.178	.240	.601	1.664	.040	.434	.007	.093	.926	.595	1.681
	MF: fairness	.404	.797	.033	.506	.613	.635	1.575	.084	.467	.012	.181	.857	.649	1.541	.333	.443	.052	.751	.453	.635	1.575
	MF: ingroup	-.076	.584	-.010	-.130	.896	.470	2.126	-.100	.347	-.022	-.289	.773	.465	2.150	.057	.324	.014	.176	.861	.470	2.126
	MF: authority	1.556	.629	.247	2.471	**.014**	.269	3.718	1.106	.373	.293	2.963	**.003**	.274	3.654	.427	.349	.129	1.222	.223	.269	3.718
	MF: purity	.703	.545	.115	1.290	.198	.336	2.973	.181	.324	.050	.559	.577	.340	2.941	.497	.303	.155	1.643	.102	.336	2.973
	Genderism	1.521	.722	.215	2.107	**.036**	.257	3.889	1.066	.429	.256	2.482	**.014**	.251	3.977	.473	.401	.127	1.179	.239	.257	3.889
QoL	Health	-.011	.036	-.018	-.296	.768	.739	1.353	-.001	.021	-.003	-.047	.963	.738	1.355	-.010	.020	-.032	-.509	.611	.739	1.353
	Crit. life events	-.255	.242	-.058	-1.054	.293	.899	1.112	-.150	.145	-.056	-1.034	.302	.900	1.111	-.111	.135	-.048	-.828	.408	.899	1.112
	F-Test and R^2^	F(20,277) = 4.769, p = .000; R2 = .256							F(20,280) = 4.642, p = .000; R2 = .249							F(20,277) = 2.894, p = .000; R2 = .173						

*Tol *Tolerance, *Sens *Sensitivity,* Big-5*: *O* Openness, *C* Conscientiousness, *E* Extraversion, *A* Agreeableness, *N* Neuroticism, *Crit* Critical, *MF*  Moral Foundations, *QoL* = Quality of life

Regression 1 revealed that a person’s profession, injustice sensitivity, moral foundation of authority, and genderism and transphobia independently predicted the overall acceptance of normality in medicine. More precisely, the more closely the participants’ profession was related to the field of medicine, the smaller the sensitivity for injustice, the higher the authority score, and the higher the genderism and transphobia score, the higher was a person’s acceptance of normality in medicine (Table 3).

Regression 2 revealed that a person’s injustice sensitivity, moral foundation of authority, and genderism and transphobia significantly predicted the acceptance of normality in the area of mental health. More precisely, the smaller the sensitivity for injustice, the higher the authority score, and the higher the genderism and transphobia score, the higher was a person’s acceptance of normality in the area of mental health (Table 3).

**Fig. 1:**
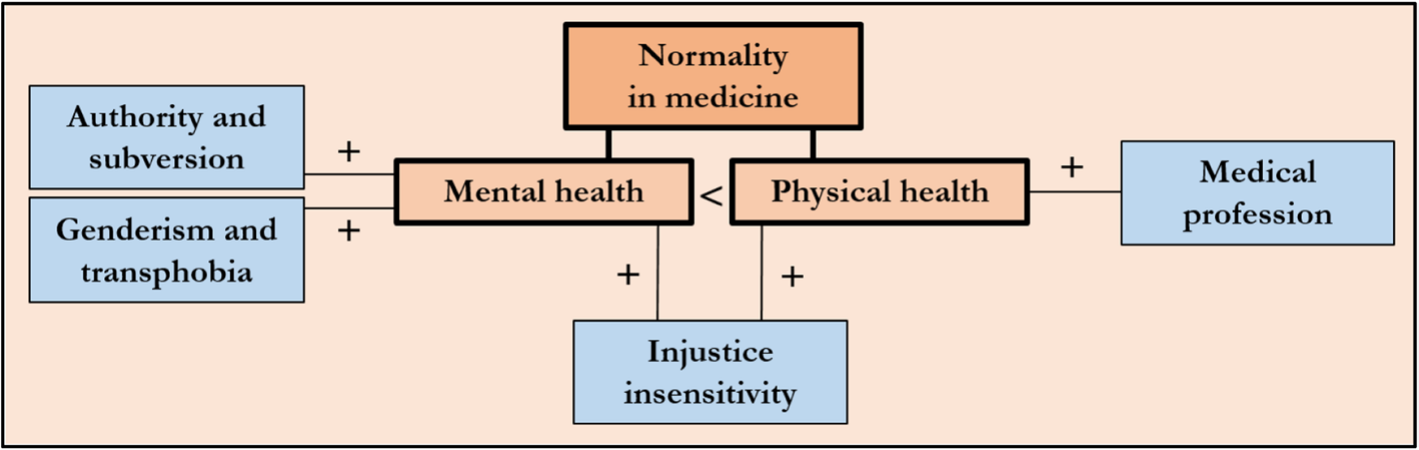
Conceptual map

Regression 3 revealed that a person’s profession and injustice sensitivity significantly predicted the acceptance of normality in the area of physical health. More precisely, the more closely the profession was related to the field of medicine and the smaller the sensitivity for injustice, the higher was a person’s acceptance of normality in the area of physical health (Table 3). Statistically significant predictors are described in more detail in Table 4. Figure 1 synthesizes the results of the three regression analyses and the paired samples t-test (mental health versus physical health) in the form of a conceptual map.

**Table 4:** Content description of statistically significant predictors

Predictor	**Description**
(Non-)medical profession	- 10-point Likert item ranging from 1 (not at all) to 10 (entirely) - Wording: “How closely is your professional background related to the field of medicine?” - Assesses the relatedness of ones’ profession to medicine
Injustice sensitivity [47]	- Measures the psychological characteristic of injustice sensitivity - Four subscales: victim’s, observer’s, perpetrator’s, and beneficiary’s perspective - Contributes to explaining social phenomena (e.g. political protest, altruism, moral courage) - Interindividual differences in how easily one perceives injustice, how strongly one reacts to it
Moral Foundation: Authority/ subversion [56, 57]	- Underlies leadership and followership (e.g. obedience, deference, respect for traditions) - At work when people interact with and grant legitimacy to modern institutions such as law courts and police departments and to bosses or respected professionals - Shaped by our long primate history of hierarchical social interactions - It helped to forge beneficial relationships within hierarchies; related emotions: respect, fear
Genderism/ transphobia [48]	- Assesses attitudinal and behavioral propensity components of anti-trans prejudice - Focuses on more severe expressions of prejudicial attitudes (e.g., viewing trans* people as immoral, disgusting, shameful)

### Exploratory moderator analyses

Since the research team hypothesized that the reasons for the acceptance of normality in the fields of mental and physical health (i.e. injustice sensitivity, authority and subversion, genderism and transphobia) are different between persons with and persons without a medical background, we conducted moderator analyses. For this purpose, three interaction terms were added to regression models 2 and 3: [1] medical profession x injustice sensitivity, [2] medical profession x authority and subversion, [3] medical profession x genderism and transphobia. None of the moderators was statistically significant, indicating that the reasons for accepting normality were not different between persons with and without a medical background.

## Discussion

Our study presents the first quantitative empirical analysis of how some people, as represented by our sample, may view normality (in medicine) and, thereby, provides hints as to which other concepts are related to it. Filling this gap is needed to deconstruct normality’s normative power and to better understand how it operates in real world environments, such as clinical practice. Our findings lend empirical support to the well-established theoretical view that normality is a normatively loaded concept. Given that the term “normal” appears 1445 times in the ICD-11, medicine has to be critically aware of its normalistic condition and the potential harms that might come along with it.

We found that the overall acceptance of normality in medicine was associated with notions of injustice, authority, discrimination, and – less surprisingly – with having a medical profession. More precisely, for the field of mental health, injustice insensitivity, genderism and transphobia, and authority were associated with a person’s acceptance of normality; and, for the field of physical health, injustice insensitivity and having a medical profession were associated with a person’s acceptance of normality.

### Normality in mental health

*Injustice sensitivity—the more sensitive to injustice, the less accepting of normality.* In the realm of mental health, the normal has been criticized as injust [15–17]. Critics of the idea of a normal mind have advocated for replacing the existing “’disability’ or ‘illness’ paradigm with a ‘diversity’ perspective that takes into account that variation can be positive in and of itself” (15,p349). In a similar vein, various movements have joined the call for the reconceptualization of some mental health disorders (e.g. autism, attention deficit hyperactivity disorder) as differences that might deviate from the mental skills of the majority of people, but that do not necessarily represent deficiencies. For example, neurodiversity and twice-exceptional (2e; i.e. gifted persons with co-existing disabilities) movements have sought to transcend the dichotomy of health vs. disorder, by emphasizing a deficit-as-difference view and a neurodiverse spectrum [15, 58–62]. These movements share the conviction that equating “difference” and “deficiency” is unjust, with some advocating for using the term “variation” instead of “difference”. This means that the abnormal should not be viewed as deficient and, ultimately, that there is no such thing as a “normality” in mental health.

Research shows that news media often portray people with mental illnesses as being violent and irrational and that such narratives have detrimental impacts on community attitudes, mental health policy, and on how these persons are treated within services and the community at large [63]. Such stereotypes and prejudices can lead to what Fricker has called “testimonial injustice” wherein people’s credibility as knowledge holders is questioned because of prejudices about them [64]. Bearing in mind that, first, (some) mental health diagnoses are perceived as unjust, and that, second, their portrayal in news media may abet further injustices, the revealed relationship between mental health related normality and injustice might be explained by a belief that the conceptualization and depiction of mental illness is unjust. That is, the more sensitive participants in our study were to injustice, the less accepting of normality in the field of mental health they were.

*Genderism and Transphobia – the more genderist and transphobic, the more accepting of normality.* Transgender and homosexual people have long been classified as being mentally ill. It was only in its most recent manual, the ICD-11, that the World Health Organization (WHO) depathologized gender incongruence. That means that gender incongruence was considered a gender identity disorder until May 2019. In addition, transgender people have been experiencing significant prejudice, discrimination, violence, and other forms of stigma (e.g. recent transgender military ban in the US) [50, 51]. In a binary system, transgender people have been stigmatized as non-normative, abnormal, and the “other”, which not only reinforces the binary beliefs of the cisgender majority, but also their power and privilege [49, 65]. Our finding that the more genderist and transphobic participants in our study were, the more accepting of normality in the field of mental health they were, provides additional support to this perspective.

*Authority and subversion – the more authoritarian, the more accepting of normality.* First it is important to underline that the inherent authority of disease classification systems does not explain this finding. Participants were not informed about the source of the respective examples of the normal in medicine (i.e. ICD-11, DSM-5) and hence their acceptance of normality in the field of mental health should not be interpreted as adherence to the medical authority of disease classification systems. Moreover, even for those participants who have recognized the source of the respective examples, such an interpretation is likely to be mistaken, because the great majority of those persons must have had a medical background, but the latter did not predict a person’s acceptance of mental health related normality. However, the mere presentation of these examples within a research setting could have been perceived as authoritarian, since researchers usually are seen as epistemic authorities.

Our finding is more likely explained by the nature of the concept of normal. The moral foundation of authority and subversion is triggered by “acts that are seen to subvert the traditions, institutions, or values that are perceived to provide stability” (56,p14). The cultural authority of established normality can be seen as such stability providing value. Furthermore, Link and Phelan describe stigma as a tool to keep other people down (e.g. racial stigmatization as a means of domination), in (e.g. enforcing social norms), and away (e.g. unhealthy persons) [66]. They use the term “stigma power” to refer “to instances in which stigma processes achieve the aims of stigmatizers with respect to the exploitation, control or exclusion of others” (65,p24). In all three cases, stigma is being used to achieve objectives that lie beneath the exercise of stigma. Similarly, the normal has been used to dominate and control people. Consequently, the normal is both a stigmatizing tool to hold people down and in, and a stability and orientation providing value. In both instances, it contains elements of hierarchical social interactions and, hence, is associated with the moral foundation of authority and subversion.

### Normality in physical health

*Injustice sensitivity—the more sensitive to injustice, the less accepting of normality.* Also in the realm of physical health, the normal has been criticized as injust [10, 13, 14]. The idea of a normal body and normal abilities imposes normative assumptions upon persons with differently abled bodies. As in the case of normality in the field of mental health, the disability-based discrimination has been addressed by various movements (e.g. disability rights movement) and legislations (e.g. Americans with Disabilities Act of 1990). They share the conviction that people with disabilities have not been experiencing equal opportunities and rights, which, fundamentally, represents injustice. The found relationship between physical health related normality and injustice might thus be explained by persons’ knowledge about the history of injustice towards persons with disabilities and by persons’ conviction that considering differently abled bodies as abnormal is unjust. That is, the more sensitive participants in our study were to injustice, the less accepting of normality in the field of physical health they were.

*Medical Profession – the “more medical” the background, the more accepting of normality.* While a person’s medical profession did not predict acceptance of normality in the field of mental health, it did so in the field of physical health. That means that persons with a medical profession themselves were as unaccepting of normality in the field of mental health as the remainder of the sample, but more accepting of normality in the field of physical health. One explanation for this finding might be that physical health as indicated by measurable physiological parameters has much more the “aura” of being scientific, objective, and based on evidence. In fact, research suggests that therapeutic pessimism exists among mental health professionals. Holding pessimistic views about the likelihood of recovery possibly mirrors skepticism surrounding the objectivity of and evidence for mental health diagnoses (psychiatric nosology), as compared to physical health [67, 68]. Another explanation might be that participants with a medical background (among which only a minority might have worked in the field of mental health) were more likely to recognize the examples of the normal in the field of physical health (i.e. ICD-11) and therefore were more likely to approve these examples as compared to examples of the normal in the field of mental health (i.e. DSM-5).

### Further considerations

It has to be acknowledged that normality is not always perceived as something questionable, but that for many people “being normal” provides stability, orientation, and safety. Of course, the underlying conformity pressure that comes along with any form of (normatively loaded) normality and that may cause fear of denormalization on the part of the individual must be critically examined [36]. Hence, besides normality as an object of scrutiny and criticism, normality can also be a “safe haven” for systematically marginalized persons who historically have been considered “different” or “abnormal” and who claim to be normal. Combating for a widening of normality, their objective is a more inclusive normality that integrates a broad(er) variety of phenomena. In fact, the German discourse theorist Link argues that in today’s dominant Western-societal strategy of how normality unfolds within society, namely Flexible Normalism, one can observe an expansion of normality, porous boundaries of normality (far off from the average), and an integration of previous abnormalities [35, 36]. To exemplify the mechanisms of Flexile Normalism, some intersex persons claim that being born with a variation of sex development is a normal and natural phenomenon. Something similar has happened in the case of homosexuality or left-handedness. In all these cases, normality has (been) expanded and the “abnormal” has become “normal”. On the contrary, in Protonormalism – the other strategy of how normality unfolds within society, normality determining norms are defined ex ante, for example by religious, political or medical authorities, and are imposed top down on individuals [35, 36].

The distinction between Flexible Normalism and Protonormalism also offers an explanation for the different attitudes towards normality as observed in our study. Normality in medicine seems to be defined by medical authorities, such as disease classification systems, and imposed on individuals. This, at least in people’s views, resembles a protonormalistic strategy. If Protonormalism has been replaced by Flexible Normalism in Western societies, as Link argues [35, 36], then a protonormalistic definition of normality in medicine, must be disapproved by persons who endorse porous boundaries and an expansion of normality. In contrast, participants with a medical background might have been educated more towards some sort of medical Protonormalism and, therefore, were more accepting of normality in the field of physical health (than persons without a medical background). Overall, participants’ acceptance of the use of normality in the area of physical health was higher than in the area of mental health. As described above, this might be explained by participants’ perceptions of physical health as being more scientific, objective, and based on evidence. It might be that medical protonormalistic boundaries between normal and abnormal physical qualities appear to be more acceptable because of better measurement.

The aforementioned benefits of normality surrounding a “safe haven” underline that – in light of the dangers related to normality – getting entirely rid of normality may not be the ideal path and, particularly for medicine, not be a realistic short term scenario. In fact, even within the debate regarding the implementation of personalized medicine, that should diminish the importance of normality in medicine due to its focus on the individual patient [27], scholars are currently asking themselves how to overcome the challenges that this new technology is posing to the definition of normal values in medicine. Identified strategies for the moment include to tailor the definition of “normal” upon the individual patients’ attributes instead across demographically diverse population, a feature that, one the one hand, underscores a distancing from viewing normality in medicine as drawing distinctions between different individuals, but, on the other hand, highlights how medicine is probably not yet ready completely discard the term [69]. The benefits and needs of the term “normal” therefore mandate to consider a broadening of normality as an instrument to mitigate the dangers of its exclusionary cultural authority. Self-evidently, an infinite expansion of normality, however, necessarily leads to a (self-)removal of normality – when everything is normal, normality disperses and qualifying something as normal becomes meaningless.

The identified associations of normality with injustice, authority, and discrimination can be integrated into a coherent conceptual framework. In people’s representations, the normal discriminates, because “it draws distinctions among people (…) in a way that is wrong” (e.g. the conviction that there is no normality and no abnormal and, thus, classifying someone as abnormal is stigmatizing) (70,p833). Such discrimination, in turn, is perceived as unjust, because it results in a situation in which not everyone have their basic needs met (e.g. intersex persons, same sex parents, children with attention deficit hyperactivity disorder) [71]. Ultimately, the normal is a tool of cultural authority enabling and subtly enacting discrimination in the first place (e.g. medical authority of classification systems, legislative authority) [2].

Lastly, none of the included personality domains was significantly associated with a person’s acceptance of normality. This is surprising, since numerous relationships between personality, prejudice, and ideological attitudes have been identified [41]. One possible explanation is that we used a short version of the Big Five inventory that might not have retained the longer versions’ predictive properties. Future research should further investigate the impact of personality on a person’s inclination to adhere to and approve the normal.

### Limitations

This study has several limitations. First, convenient online sampling might have resulted in an overrepresentation of individuals with an interest in or a strong opinion on the topic, respectively. Related to this, recruiting participants through professional contacts might have led to an overrepresentation of participants who assess their political attitudes as left. Consequently, we fully acknowledge and embrace the limitations of our study related to sampling. Second, the sample is not representative of the global population, but represents a mainly WEIRD (western, educated, industrialized, rich, democratic) sample [72], which limits the generalizability of our results. Third, facing a dearth of scales measuring a person’s notion or acceptance of normality, the research team created new normality scales. Nevertheless, the respective scales were shown to be internally consistent and used examples of disease classification systems which unarguably represent real-life examples of the normal in medicine. Lastly, it has to be noted that measuring personality- and morality-related constructs necessarily reduces the complexity of underlying concepts. For example, injustice sensitivity, hence, cannot fully capture the concept of (in)justice. Nonetheless, the identified relationships between measured constructs provide first hints towards conceptual relationships of normality.

## Conclusions

Undoubtedly, medicine reifies the normal for the fields of mental and physical health. However, it has been shown that the normal – in medicine and in other disciplines – divides people into subpopulations of asymmetric social power (those who are normal and those who are abnormal) and, thereby, risks to perpetuate systems of oppression and inequality (e.g. historically marginalized communities). If we envision “a world in which everyone can live healthy, productive lives, regardless of who they are”, as expressed in the WHO’s vision statement [73], medicine has to confront itself with its own contribution to the augmentation of social inequalities through the excessive reliance on the concept of normality. Ultimately, social inequalities are important determinants of health. Moreover, what is considered normal certainly has implications for clinical practice, both at an individual and at a policy-level [8]. It directly affects patients.

Our findings reveal important insights into the subject of normality. First, they illustrate that the normal is indeed a normative concept. The concept of normal in medicine, in people’s views, is associated with injustice, authority, and discrimination. This echoes Canguilhem’s words: “to normalize is to impose a requirement on an existence” (6,p239). Second, since the normal operates similarly in different normality fields [35, 36], it can be argued that our findings are generalizable beyond medicine to other fields of normality. Third, our analysis suggests that laypersons are not as accepting of the normal in medicine as health professionals. The latter appear to have internalized the idea of normality in the field of physical health (at least more than the former). Fourth, since the discipline of medicine is widely recognized and respected, it may represent a major driver for the idea of normality in general. Finally, by empirically elucidating the conceptual relationships between normality in medicine and other variables, we provide points of leverage to deprive normality of its normative power.

Lastly, only injustice sensitivity was associated with both the acceptance of normality in the field of mental and of physical health. This points towards a perception of the normal in medicine as an unjust concept. Injustice, however, is not the mere absence of justice, but should be studied as an independent phenomenon in its own right and with a particular emphasis on the victim’s sense of injustice to arrive at a more complete account of injustice (e.g. its social character) [74]. In conclusion, acknowledging that the use of normality in medicine, sometimes, is unjust (discriminatory, and authoritarian) in the first place, the research community has to bring about more studies that center the lived experiences of those who are being systematically marginalized because they are deemed abnormal. Such research can inform the creation and implementation of culturally safe environments. For medicine, this is needed to first do no harm.

## Supplementary Information

**Additional file 1. **

## Data Availability

The dataset generated and analyzed during the current study is available in the Swiss FORS repository, https://forscenter.ch/data-services/data-at-fors/. The dataset used during the current study is available from the corresponding author on request.

## Abbreviations

ICD: International Statistical Classification of Diseases and Related Health Problems
DSM: Diagnostic and Statistical Manual of Mental Disorders
EKNZ: Ethikkommission Nordwest- und Zentralschweiz
HDI: Human Development Index
WEIRD: Western, educated, industrialized, rich, and democratic
