# Supplementary material for: Normality in medicine: an empirical elucidation
Source: Philos Ethics Humanit Med. 2022 Dec 21;17:15. doi: 10.1186/s13010-022-00127-z (PMC9768910; doi:10.1186/s13010-022-00127-z)
Supplement: Supplementary file 1 — Additional file 1. [file 13010_2022_127_MOESM1_ESM.docx]

**Appendix**

***Table***. Corrected Item-Total Correlation for normality subscales

| Items mental health | Correlation | Items physical health | Correlation |
| --- | --- | --- | --- |
| normal fluency and time-patterning of speech | .515 | normal pregnancy | .487 |
| abnormality of beliefs, thinking, and perception | .595 | abnormality of gait and mobility | .539 |
| normal pattern of learning academic skills | .567 | normal physiological development | .542 |
| abnormal social  approach | .560 | abnormal bowel sounds | .468 |
| normal level of intellectual functioning | .626 | normal head movements | .499 |
| abnormality of emotional or cognitive processing | .572 | abnormality of heartbeats | .390 |

**Assumptions check for regression analyses**

Cook’s distance indicated that the data contained no outliers (highest values for regression 1 = .065, regression 2 = .042, regression 3 = .059), suggesting no individual cases were unduly influencing the model. Collinearity statistics (Tolerance, VIF) indicated that multicollinearity was not a concern (table 3). Durbin-Watson test indicated that the data met the assumption of independent errors (Durbin-Watson for regression 1 = 1.763, regression 2 = 1.764, regression 3 = 2.041). For all three regressions, histograms of standardized residuals indicated that the data contained normally distributed errors, as did the normal P-P plot of standardized residuals, which showed points that were not entirely on the line, but close. For all three regressions, scatterplots of standardized predicted values showed that the data met the assumptions of homoscedasticity and linearity. The data also met the assumption of non-zero variances.
